# A Novel RFID Authentication Protocol Based on Reconfigurable RRAM PUF

**DOI:** 10.3390/mi12121560

**Published:** 2021-12-15

**Authors:** Qirui Ren, Xiangqu Fu, Hao Wu, Kaiqi Yang, Dengyun Lei, Guozhong Xing, Feng Zhang

**Affiliations:** 1 Key Laboratory of Microelectronics Device and Integrated Technology, Institute of Microelectronics of the Chinese Academy of Sciences, Beijing 100029, China; renqirui@ime.ac.cn (Q.R.); fuxiangqu@ime.ac.cn (X.F.); wuhao@ime.ac.cn (H.W.); yangkaiqi@ime.ac.cn (K.Y.); gzxing@ime.ac.cn (G.X.); 2 The No.5 Electronics Research Institute, Ministry of Industry and Information Technology, Guangzhou 510610, China; leidengyun@163.com

**Keywords:** radio frequency identification technology (RFID), resistive random access memory (RRAM), physical unclonable function (PUF), reconfigurability, security authentication protocol

## Abstract

Radio frequency identification technology (RFID) has empowered a wide variety of automation industries. Aiming at the current light-weight RFID encryption scheme with limited information protection methods, combined with the physical unclonable function (PUF) composed of resistive random access memory (RRAM), a new type of high-efficiency reconfigurable strong PUF circuit structure is proposed in this paper. Experimental results show that the proposed PUF shows an almost ideal value (50%) of inter-chip hamming distance (HD) (µ/σ = 0.5001/0.0340) among 1000 PUF keys, and intra-chip HD results are very close to the ideal value (0). The bit error rate (BER) is as low as $3.8\times 10^{-6}$ across one million challenges. Based on the RRAM PUF, we propose and implement a light weight RFID authentication protocol. By virtue of RRAM’s model ability, the protocol replaces the One-way Hash Function with a response chain mutual encryption algorithm. The results of test and analysis show that the protocol can effectively resist multiple threats such as physical attacks, replay attacks, tracking attacks and asynchronous attacks, and has good stability. At the same time, based on RRAM’s unique resistance variability, PUF also has the advantage of being reconfigurable, providing good security for RFID tags.

## 1. Introduction

Radio Frequency Identification (RFID) is a technology that uses wireless radio frequency for contactless communication to achieve target recognition. A complete RFID system is generally composed of a reader, a tag and a server/database. With the wide application of RFID technology, the channel security between reader and RFID tag has been paid more and more attention. Since the communication between the reader and the electronic tag is implemented through the wireless air interface, an attacker can easily monitor, intercept and falsify all information during communication.

In general, RFID tags are considered as resource constrained devices; accordingly, it is always feasible to use lightweight cryptographic primitives in designing an anonymous authentication protocol for RFID system [1]. Therefore, most of the RFID authentication protocols use a symmetric-key system such as a hash function. On the other hand, physically unclonable functions (PUFs) [2] are clone proof, cost efficient and resistant to various physical attacks, which introduce random physical verifications into the micro structure of IC, to make it unique. PUFs are basically ICs, which use their internal structures to provide one-way function that cannot be duplicated. The PUFs are easy to construct with a few numbers of gates [3] and their outputs are difficult to predict but easy to evaluate. This makes them a good choice for use as primitive security for an RFID system.

Recently, emerging non-volatile memory (NVM) based PUFs have been proposed [4], including phase change memory (PCM) PUFs [5], spin torque transfer magnetic random access memory (STT-MRAM) PUFs [6] and resistive random access memory(RRAM, or memristor) PUFs [7,8,9,10,11,12,13], such as Cross Bar PUF, RRAM PUF, and EPUF. However, most still suffer from some issues, mainly reliability, reconfigurability, and extensive integration cost. It should be noted that the NVM PUF may not follow the canonical definition of PUF, but it is more similar to a true random number generator with a (secure) NVM. Most of these prior works are based on simulations [13] or a single device measurement, which usually could not accurately reflect the statistics of variability and reliability in the memory arrays. To date, there are limited experimental data available in the literature about NVM based PUFs’ characteristics at the array-level. In [5], PCM PUFs were experimentally evaluated on 1 Mb arrays, showing that the mean value of inter-Hamming distance (HD) is around 30% without hash and intra-HD can be up to 10%. Many of the prior works [7,8] use the probabilistic switching of RRAM as the entropy source. However, the cycling endurance degradation will eventually limit its lifetime, and the active switching in each cycle consumes much more energy than the static read-out of resistance. In [12], although using RRAM resistance variability can achieve a high reliability, the weak PUF may not perform particularly well in terms of security.

On the basis of the above research, this paper proposes a new type of RRAM PUF circuit structure and a lightweight RFID authentication protocol based on this structure. The circuit improves the computational efficiency of the PUF circuit and expand the challenge response space. This protocol can be applied not only to RFID systems that include RRAM PUF, but also to other RFID systems that include reliable PUF models, which are expected to provide more comprehensive protection for RFID.

The remainder of this paper is arranged as follows: In Section 2, the works related to PUF-based security protocols are introduced. In Section 3, we describe the specific implementation method of the RRAM PUF circuit. In Section 4, we introduce the details of the proposed authentication protocol. In Section 5, the experimental results of the RRAM PUF circuit and the protocol characteristics’ analysis are presented. Finally, the paper is summarized in Section 6.

## 2. Research on RFID Security Mechanism Based on PUF

Similar to the uniqueness of finger prints or irises, PUFs uniquely identify targets by amplifying the subtle physical differences that occur during chip fabrication. In 2002, Pappu [14] first designed and implemented optical PUF. Later, with the demand for light weight encryption and decryption authentication algorithms, more PUF implementation methods have emerged [15,16,17,18,19,20,21]. Through the analysis and summary of PUF, we can draw some basic security attributes about PUF. Here are some of them:

(1) Non-clonality, even for any two PUF modules F having the same internal structure, given an input x, the difference between their outputs *F*(x) and *F*${}^{\prime }$(x) is greater than a determinable distance. The distance between *F*(x) and *F*${}^{\prime }$(x) is called the inter-chip Hamming Distance (inter-HD);

(2) Unpredictability, in the case where a plurality of challenge response pairs (CRPs) of the PUF module are known to be (x${}_{i}$, *F*(x${}_{i}$)) (i = 1, 2, *…*, q). It is difficult to predict *F*(x) for any x (x${}_{1}$, x${}_{2}$, *…*, x${}_{q}$);

(3) Robustness, for any PUF module, the *F*(x) and *F*${}^{\prime }$(x) obtained within a certain error range are always equal after the same excitation x is input twice. The difference between *F*(x) and *F*${}^{\prime }$(x) is called intra-chip Hamming distance (Intra-HD). The size of intra-HD and its fluctuation with the working environment are important criteria to measure the reliability of a PUF.

Although PUF has many security attributes, most of the RFID systems based on PUF are still affected by security threats such as replay attack, asynchronous attack and tracking attack. On this basis, domestic and foreign scholars have proposed many RFID authentication schemes based on PUF. We have studied and analyzed some of them.

Suh [3] firstly utilized PUF in RFID systems, where each response of the PUF tag is stored in the backend database, and the responses will be extracted from the database during authentication. If the generated response of the tag is consistent with that in the database, the authentication is successful. Note that this protocol has realized one-way authentication of the tag. In 2016, Zhu et al. [22] presented a novel PUF based LMAP+ protocol. It is an effective solution to resist the heuristic attack strategy in [23]. The protocol is also immune to tracing attack and asynchronous attack. In [24], a double PUF based bidirectional RFID identity authentication protocol is proposed, aimed at providing authentication for IC, such as a low-cost RFID system. In 2019, Zheng et al. [25] proposes a lightweight RFID authentication protocol based on PUF, which uses the machine learning algorithm and string-matching algorithm, and presents a new mechanism of PUF challenge generation. A PUF-based anonymous RFID system ownership transfer protocol is presented in [26], using the anonymous RFID system and some encryption schemes to protect the location privacy of tags and the problem of information privacy during transfer. In [27], a lightweight RFID security authentication protocol based on a cloud server is studied. Using physical unclonable function (PUF), permutation, string matching and other lightweight encryption algorithms to reduce hardware resource consumption. However, among the many proposals currently proposed, there are heavy computations in complex updating algorithm and many data interactions between tag, reader and backend database as well. Although security is improved, it causes large hardware overhead. Besides, many protocols cannot resist multiple attacks and have high cost.

Based on this, this paper proposes an RFID authentication protocol based on the RRAM PUF. The protocol uses a special RRAM PUF model and a multi-level response computer system to complete the authentication information encryption between the reader and the tag. During the authentication process, the tag side consumes only a small amount of computing resources; it uses a real-time shared key exclusive OR (XOR) method to quickly calculate the unique identification number of the tag, realizing the rapid retrieval of the tag by the reader; the unique error correction processing method enables the error correction of the PUF response without leaking the authentication information, which improves the reliability of the protocol authentication process. Finally, based on formal analysis, logic synthesis and test, this article proves the protocol can resist multiple attacks and has good safety and stability.

## 3. RRAM PUF Circuit

**Figure 1:**
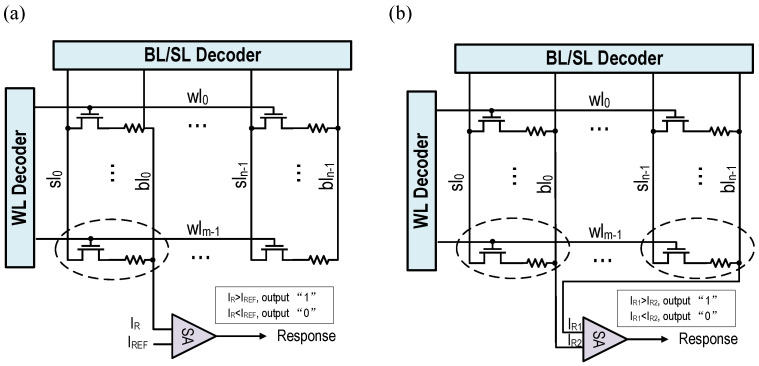
(**a**) Traditional RRAM-based weak PUF. (**b**) Traditional RRAM-based strong PUF.

The typical PUF structure based on traditional RRAM is shown in Figure 1a,b. Unlike other PUFs that generate random output bits using subtle differences in the manufacturing process, the RRAM implements the PUF function by its discrete resistance distribution formed after erasing. Its physical mechanism is generally attributed to the formation and rupture of conductive filaments formed by oxygen vacancies between two metal electrodes. Because of the randomness of the generation and the disappearance of oxygen vacancies, the shape and size of conductive wires vary randomly in different RRAM cells or even in different periods of the same RRAM cell [11]. Then, a random resistance distribution is formed between different RRAM cells. If a suitable reference current is set, a 0 or 1 can be read out.

When the PUF with the structure of Figure 1a is working, the PUF challenge input is used as the address of the relevant decoder and an RRAM cell is selected, and then the read current I${}_{R}$ of the cell is compared with the reference current I${}_{\mathrm{REF}}$, and the sense amplifier (SA) can output a 1-bit PUF response. When a multi-bit PUF response needs to be generated, the operation needs to be repeated many times. This structure is limited by the number of RRAM units, can only generate limited challenge response pairs (CRPs), and has low work efficiency. It is a typically weak PUF and cannot be directly applied to scenarios such as identity authentication. Different from the structure in Figure 1a, the scheme in Figure 1b selects two RRAM cells according to the challenge bit, and then the resistance of the two cells is compared as the output of the PUF response. Due to the difference in response calculation methods, this scheme constitutes a strong PUF circuit, but when a multi-bit response needs to be generated, the read operation must be repeated multiple times, and the work efficiency is still low.

**Figure 2:**
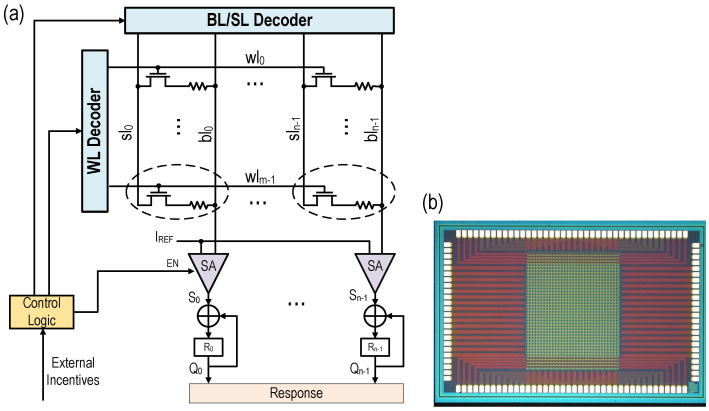
(**a**) Structure of proposed RRAM PUF circuit. (**b**) Microphotograph of the fabricated RRAM array chip.

In order to improve the computational efficiency of the PUF circuit and expand the challenge response space, this paper proposes a new type of PUF circuit based on RRAM. As shown in Figure 2, the overall structure of the PUF mainly includes PUF control logic modules, 32 rows × 32 columns of RRAM memory cells, 32 word lines (WL), 32 bit lines (BL), 32 source lines (SL), 32 sensitive amplifiers (SA), and 32 exclusive OR (XOR) gates and 32 D flip-flops, where each RRAM memory cell is composed of an N-type transistor and a resistive memory cell. Among them, the PUF control logic module receives the excitation signal of the main system (including PUF response calculation request, 32-bit PUF challenge, read/write request signal and corresponding address, etc.), and generate the decoder control signal, address and other read/write control signals of the RRAM circuit according to the above signals, and complete RRAM configuration, read/write, PUF response calculation and other functions.

The traditional memory PUF generally uses the PUF challenge as the memory address and selects the PUF unit, and then reads the stored value of the corresponding unit as the PUF response. For a multi-bit response, it needs to be read multiple times. The proposed RRAM-PUF circuit generates the address signal of the word line decoder according to the 32-bit challenge, and judges the challenge bit by bit. If challenge [k] = 1 (k = 0, 1, 2, *…*, 31), then control the word line decoder to select the word line WL${}_{k}$, and then read out the values stored in all RRAM cells in the k-th row and perform an XOR calculation with the flip-flop output of the corresponding column. Save the calculation result in the trigger again. If challenge [k] = 0, no read operation is performed. By repeating the above operation 32 times, a 32-bit PUF response can be obtained. Since the bit line is turned on once every time, the accuracy of the output data is improved during the read operation, and at the same time, the 32-bit challenge can be used to generate 2${}^{32}$ CRPs, which greatly improves the calculation efficiency and expands the challenge response space. Moreover, since the RRAM PUF has different resistance distribution in different reset cycles, it can be reconfigured repeatedly.

## 4. Protocol Details

In view of the shortcomings of existing RFID authentication protocols based on PUF in terms of stability, security and cost, we propose a better solution combining the unique advantages of RRAM PUF. The hardware structure of the protocol is very simple. The tag side consists of a PUF module, an error correction algorithm and some XOR gates. In addition, a “native model” of the PUF is saved on the database side. The protocol is divided into three parts: registration phase, certification phase and reconfiguration phase. The first two stages are necessary and the last one is optional. For the convenience of description, the database and reader will be collectively referred to as readers. We will describe the protocol in detail below.

### 4.1. Registration Phase

**Figure 3:**
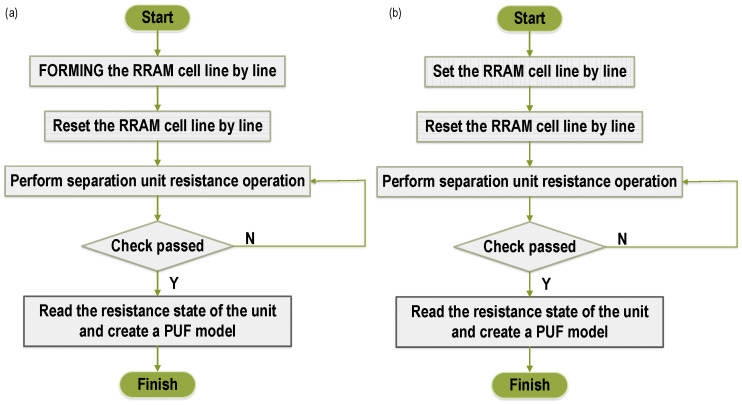
(**a**) RRAM PUF configuration process. (**b**) RRAM PUF reconfiguration process.

At this stage, the Registrar initializes the tag chip. The configuration process is shown in Figure 3a. Perform an initial forming operation on the newly prepared RRAM, so that a stable conductive path is formed inside all RRAM memory cells. At this time, the RRAM cell device presents a low resistance state (LRS). Since the resistance values of different RRAM cells present a more discrete distribution in the high resistance state, a reset voltage of the same pulse width is then applied to all RRAM memory cells row by row, causing the conductive filaments inside the RRAM cell to break and turn into high resistance state. Then, an appropriate reference current I${}_{\mathrm{REF}}$ is set, so that the RRAM cells whose read data is “1” account for about 50% of the total number of cells. (The read operation is specifically that when the read current of the selected RRAM cell is greater than the reference current, the read data is “1”, and when the read current is less than the reference current, the read data is “0”.) After completing the above steps, perform the separation resistance operation, specifically applying the set voltage to the RRAM cell whose read data is “1”, so that the selected cell turns into a low resistance state; applying reset voltage on the RRAM cell whose read data is “0”, so that the selected cell turns into a high resistance state. The step of separating the resistance described above can improve the stability of data output.The configuration ends until all RRAM cells have successfully completed the configuration and can read stable data normally. After completing the above steps, the relative resistance of each cell can be read out and the “native model” RS of the PUF module can be created, and then stored at the legitimate database. The model here is essentially different from the model mentioned in [10]. In [10], the registrar established an approximate model for each PUF module through machine learning, so there is an inevitable error rate. On the other hand, the PUF module itself also has intra-HD, so the final response of the model may be very unreliable. The model RS in this paper, when inputting an arbitrary challenge, theoretically produces the same response as the entity of PUF.

In order to reduce the bit error rate of the output, after the configuration is completed, it is also necessary to set the relatively low resistance cell to LRS to increase the resistance difference (similar to the “Forming” operation, “Set” causes the conductive filaments to form inside the RRAM cell by applying an electric field, but the required voltage is usually much smaller than “Forming”).

The reader side stores information <ID’, K${}_{i}$, K${}_{i-1}$, S, RS> for each tag, where ID is the unique identifier of the tag. K${}_{i}$ and K${}_{i-1}$ are keys shared with the tag and will be updated after each authentication. When initializing, K${}_{i}$ = K${}_{i-1}$. S is the authentication completion identifier, S = 1 means that the corresponding tag last round authentication is successful, and it is not attacked, and S = 0 means the last round authentication failure. RS is a PUF native model. The tag side saves information <ID, K, M${}_{0}$>, in which ID is the unique identification number, K is the key shared with the reader side, and is updated after each authentication. M${}_{0}$ is the seed of random number.

### 4.2. Certification Phase

**Figure 4:**
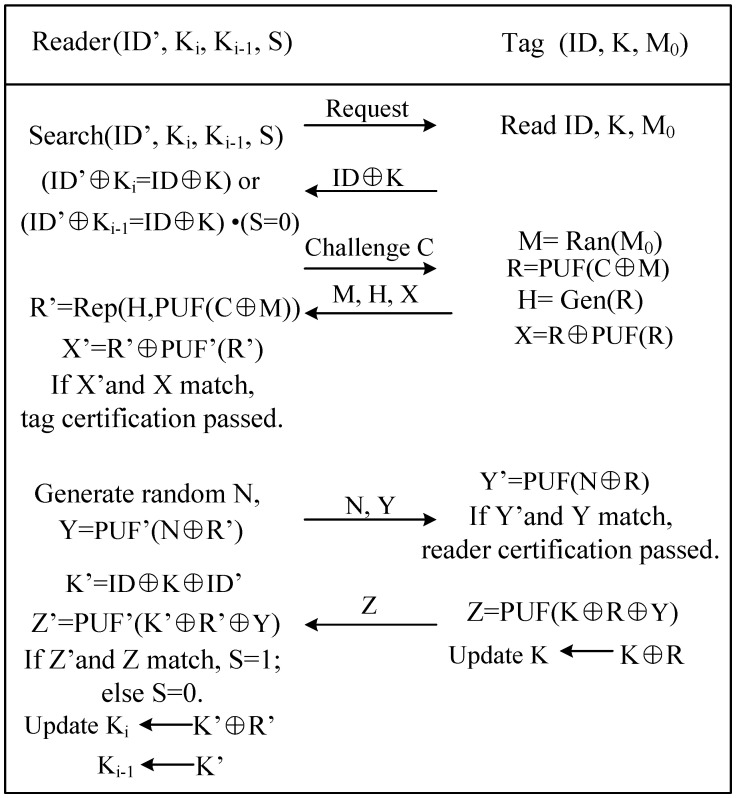
The flow chart of the authentication protocol.

(a) The reader sends an authentication request. After the tag receives the authentication request, it reads the ID and K stored in the memory and sends the ID ⊕ K as the response information to the reader;

(b) For each tuple <ID’, K${}_{i}$>, the reader calculates ID’ ⊕ K${}_{i}$ and if it matches ID ⊕ K, the tag is successfully recognized. If it does not match, the reader enters the next round of search, that is, to each tuple <ID’, K${}_{i-1}$, S> continue to calculate ID’ ⊕ K${}_{i-1}$. If it matches ID ⊕ K and S = 0, the tag is successfully identified and the protocol enters the next stage. Otherwise, the reader no longer responds and terminates the protocol;

(c) The reader generates a random number C and sends it to the tag as a Challenge. To prevent replay attacks, C here should avoid duplicate numbers, which can be generated using a TRNG or PUF module;

(d) After receiving the C, the tag reads the random number seed M${}_{0}$ and generates a random number M = Ran(M${}_{0}$). In order to reduce the area, the M here is generated by using the PUF module, and its detailed generation method is M${}_{1}$ = PUF(M${}_{0}$), M = M_1_ ⊕ PUF(M${}_{i})$. Then calculate R = PUF(C ⊕ M), X = R ⊕ PUF(R), and generate the error correction code H = Gen(R) of R, and finally send M, H, X to the reader. In addition, the random number seed M${}_{0}$ is updated to M${}_{1}$ and written to the memory;

(e) After the reader receives M, Hand X, the decoder using the error correction algorithm recovers R${}^{\prime }$ = Rep(PUF${}^{\prime }$(C ⊕ M), H), and then calculates X${}^{\prime }$ = PUF${}^{\prime }$(R${}^{\prime }$) ⊕ R${}^{\prime }$ using the PUF model. If X and X${}^{\prime }$ are within the error range, the tag is considered legal, then the reader generates a random number N, computes Y = PUF(N ⊕ R${}^{\prime }$), and sends N and Y to the tag. Otherwise, the protocol is terminated;

(f) The tag calculates Y${}^{\prime }$ = PUF(N ⊕ R). If Y${}^{\prime }$ and Y are within the error range, the reader passes the authentication, the tag updates K = K ⊕ R, and Z = PUF(K ⊕ R ⊕ Y) is sent to reader. Here Z is used as the final acknowledgment signal to detect man-in-the-middle attacks;

(g) The reader calculates K${}^{\prime }$ = ID ⊕ K ⊕ ID${}^{\prime }$, Z${}^{\prime }$ = PUF${}^{\prime }$(K ${}^{\prime }⊕$ R ${}^{\prime }⊕$ Y), if Z${}^{\prime }$ matches Z, then sets S = 1; if Z${}^{\prime }$ does not match Z or responds timeout means that an error or an attack occurred during the transmission, and S = 0 is set at this time. Finally, the reader updates K${}_{i}$ = K${}^{\prime }$ ⊕ R${}^{\prime }$, K${}_{i-1}$ = K${}^{\prime }$. The protocol flow is shown in Figure 4.

### 4.3. Reconfiguration Phase

The PUF module is reconfigurable based on the randomness of the RRAM cell’s own resistance after each erase. The overall step of the reconfiguration phase of the protocol is not much different from the registration phase. The difference is that the first step is changed from the “Forming” operation to the “Set” operation. The flow is shown in Figure 3b. Reconfigurability brings more flexibility to RFID tag security solutions. For example, we make the worst assumption, that if an attacker obtains all the CRPs of the tags stored in the database, this is almost catastrophic and irreversible for RFID systems of other solutions. But for the RFID authentication system using RRAM PUF, the tag can be reconfigured by the registrant and the RS in the database can be updated at the same time.

## 5. Experimental Results Analysis

### 5.1. RRAM PUF Test Results

**Figure 5:**
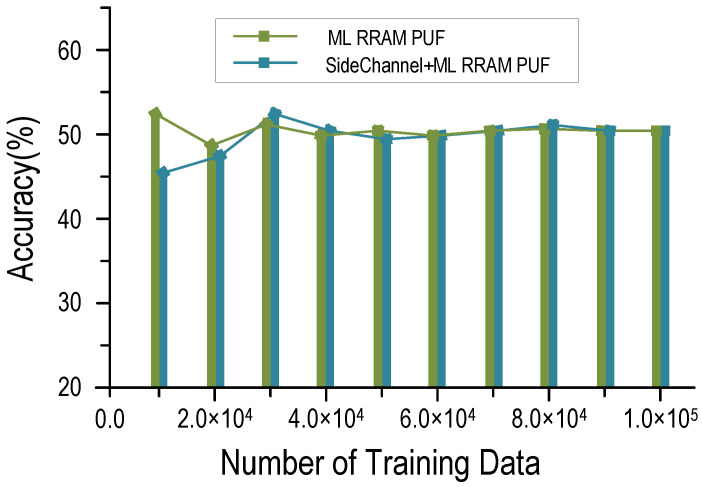
The prediction accuracy for RRAM PUF.

Attackers can use ML algorithms to map out the circuit structure of PUFs by collecting and analyzing large amounts of CRPs. The authors of [16] provide an elaborate analysis of ML attacks modeling and achieve good accuracies on various strong PUFs. We ran a simulated ML attack on the RRAM PUF. A total number of 10${}^{5}$ CRPs are used, with 8 × 10${}^{4}$ for training and 2 × 10${}^{4}$ for testing. As shown in Figure 5, the prediction accuracy for RRAM PUF is close to the ideal 50% under machine learning and side-channel attack, which means that attackers fail to make an effective prediction. The accuracy depends on the suitability of the model and can be increased with more training data. Other ML models may exist that can achieve higher accuracy when mapping out the structure of PUF in this work. An ideal strong PUF immune to ML attacks has a 50% bit accuracy when modeled and predicted in any way. Under the correlation-based power analysis attack, the number of traces to successfully analyze RRAM PUF is more than $1\times 10^{7}$, 20 times as many as that of CMOS PUF.

**Figure 6:**
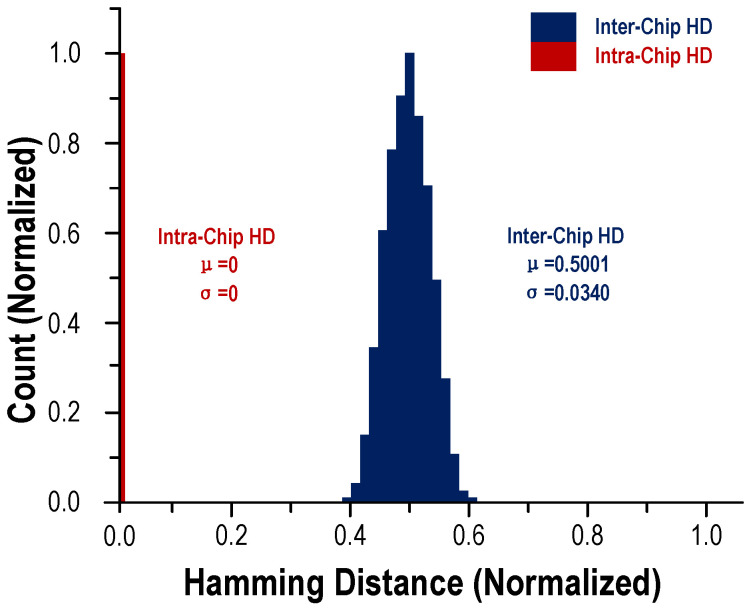
Distribution of inter-HD and intra-HD of 32-bit responses.

Two important indicators to measure the quality of a PUF are uniqueness and reliability. The former is measured by the inter-hamming distance (inter HD) of the PUF, which requires that the response generated by the PUF is sufficiently different for different challenges. If the uniqueness of PUF as an encryption function is insufficient, it proves that the output has a preference for different inputs, and this preference can easily become a breakthrough for attackers. The latter is measured by PUF and intra-hamming distance (intra HD), which requires that the PUF output response to the same challenge at any time is exactly the same even if the environment changes. Generally, in the design of PUF, in order to reduce the complexity of circuit design, it is stipulated that half of the bits in the challenge sequence are 1 and the other half are 0. For the convenience of discussion, the selected sequence assumes that the challenge sequence is 32 bits, of which 16 bits are 1, and the remaining 16 bits are 0. In the case of selecting a specific row, the sum of the currents of the selected components in each column is obtained. Since half of the address sequence is 1 and half is 0, exactly 16 RRAM devices are selected in each column. Since the components in each column are written with random values in advance, theoretically, the sum of their currents is also random. For the convenience of discussion, it may be assumed that the current flowing through the low-resistance component is a large current, and its value unit is 1, and the current flowing through the high-resistance component is a small current, and its value is 0. For each column of the selected 16 components In other words, the number of components with a high resistance state and the number of components with a low resistance state are random, and different challenges will select different 16 devices. Assuming that the resistance values of the selected 16 components are all high, the sum of the currents of the RRAM devices in this column is 0; if the resistance values of the 16 selected components are all low, the sum of the currents is 16. The sum of these large currents is 16. It is not difficult to obtain that when configured as PUF in this way, the column current is in accordance with the normal distribution, and its symmetry axis is 8; that is, for the 16 RRAM devices selected in each column, the average values of the current and the expectation are equal to 8. If you compare the current of each column with a reference current of 8, you can get a single-bit random number 1 or 0 in each column, and compare the currents of the 32 columns with the reference current to get a random 32-bit This sequence is the response corresponding to the received challenge, which can be used for the verification of the communication parties and the encryption of data. By polling PUF with 1000 different challenges, the response under each challenge can be obtained, and the Hamming distance between the slices can be calculated. The data show an almost ideal value (50%) of inter-chip hamming distance (HD) (µ/σ = 0.5001/0.0340) among 1000 PUF keys, which proves that the PUF has good uniqueness. Regarding reliability, by entering the same challenge into the same PUF instance under different test conditions, and observing whether the output is consistent, PUF reliability data can be obtained. In the actual test, the PUF designed in this article is for the same input under different conditions, and the BER is as low as $3.8\times 10^{-6}$ across one million challenges based on PUF reconfiguration (BER = 123/32 Mbit = $3.8\times 10^{-6}$), the intra-chip HD results are very close to the ideal value (0), showing near-ideal reliability. Its better reliability is also attributed to the stability and low power consumption characteristics of resistive storage. The test results are shown in Figure 6.

As for randomness, it is further evaluated using the NIST test suite. The R-SPUF has passed all the NIST security test items, with the average *p*-value ≫ 0.01 (criteria value), shown in Table 1. In Table 2, we present a comparison between the previous works and the contributions of this study.

### 5.2. Protocol Characteristics Analysis

A secure and reliable RFID authentication protocol must consider three factors according to its priority: reliability, security and low cost. In the design of the protocol, the current main stream attack methods are taken into account, and an effective solution is proposed. On the premise of ensuring reliability, the use of unnecessary hardware is reduced as much as possible. Below we will conduct a detailed analysis.

#### 5.2.1. Reliability Analysis

The reliability of the PUF-based RFID authentication protocol is reflected in the error acceptance rate and error rejection rate of the PUF [32]. On the one hand, this can be attributed to inter-HD and intra-HD of PUF. According to recent studies, RRAM PUF has a good performance in this respect. On the other hand, from the perspective of the protocol, X = R ⊕ PUF(R) in the third step, in which the first order response R is processed by the error correcting code. Depending on the non-clonality and avalanche effect of the PUF, only a perfectly matched PUF model can recover it and produce the correct PUF(R${}^{\prime }$) and X${}^{\prime }$, so the probability of accepting the wrong PUF by authentication is extremely low. Due to the use of the error correction code, the protocol can also resist a certain degree of bit error rate, ensuring that the reader will not reject the correct PUF. In addition, the protocol also considers errors and out-of-step problems that may occur when the key K is updated, which we will cover in the next section.

#### 5.2.2. Security Analysis

We analyzed the ability of the protocol to defend against current main stream attacks, as follows:

(1) Physical Attack

Assuming that an attacker can destroy the tag and access the tag’s non-tamper-proof memory to obtain information such as ID, K, M${}_{0}$, etc. On the one hand, the key K only plays the role of encrypting the tag ID, and cannot determine the response information of the tag in authentication. On the other hand, although M0 is the seed of random number of tags, M${}_{0}$ and random number M are not CRP. In addition, the subsequent X, Y, Z authentication information is encrypted based on R and RS. According to the physical unclonable characteristics of PUF, attackers cannot get R and RS from the existing information. Therefore, the information that attackers cannot get through physical attacks has any impact on authentication.

(2) Replay attack

Firstly, the tag is analyzed. From the fourth step, we can know that the tag will update the random number seed M${}_{0}$ after each authentication, so the tag will produce different random number M for each authentication, and the R, H, X based on M will naturally be different. Even if an attacker obtains the previous authentication interaction information using a wiretapping attack, it cannot perform a replay attack on the tag. Meanwhile, in order to defend against the attacker’s replay of the previous authentication message to achieve the purpose of spoofing the reader, the key is that the same challenge does not occur every time the authentication is performed. In the third step, we pointed out that a TRNG or PUF module can be integrated on the reader side. Assuming that the reader has sufficient computing and storage capabilities, there are many ways to meet the requirements. Therefore, the replay attack against the reader is also invalid.

(3) Tracking attack

Tracking attack is a threat to most passive RFID tags at present. Attackers can track tags through the ID number of tags and other information, so as to obtain the location privacy information of tag holders. They can also eavesdrop on the authentication information of the specified tag for many times, and then seek the possibility of cracking the information.

Currently, the ID numbers of tags in most protocols are sent in plaintext or simple encryption. That is to say, attackers can easily get these invariable ID numbers of tags and implement tracking attacks on tags. Although some RFID authentication protocols propose the method of updating tag ID number after each authentication, most of them have two problems, which lead to the inapplicability of the protocol: (a) The protocol is vulnerable to asynchronous attack, which makes the ID number of reader side and tag side asynchronous. (b) The protocol does not take into account the influence of intra-HD, which results in discrepancies between the updated information of reader and tag.

The protocol proposed in this paper solves the problem by using the following methods: firstly, the ID number of the tag is encrypted with the shared key, and then the reader and tag end use R${}^{\prime }$ and R to update the key respectively after each round of authentication, which prevents the tracking attack. Secondly, in the fourth step, the protocol uses error correction algorithm to ensure the consistency of R and R${}^{\prime }$, avoiding the impact of error rate on key update. Finally, the reader sets an authentication completion identifier S for each tag, while retaining the key before the tag is updated. Assuming that an attacker intercepts or tampers with Y and Z, resulting in protocol failure, the reader can retrieve the tag and restore the key in the next authentication, regardless of whether the tag updates the key K or not.

(4) Machine learning attack

At present, most strong PUFs generate a large number of incentive response pairs through repetitive hardware units. Attacker scan infer their correlation through machine learning and create PUF models. In the related literature, an attacker only needs to collect one thousand CRPs to create a model with an accuracy rate of 90% [11]. In the traditional encryption method, the Hash function is generally used to encrypt the PUF response to avoid the attacker getting CRPs. However, even the simplified Hash module requires at least 1700 gates to implement. In this protocol, combined with an error correction algorithm, the response chain generated by PUF is used to encrypt each other, that is, X = R ⊕ PUF(R). The reader can recover R by using RS and error correction code H, and subsequent authentication messages have R participation, but the attacker cannot infer R only from H. Therefore, the protocol can resist machine learning attacks.

#### 5.2.3. Protocol Comparative Analysis

In addition to the aforementioned attack methods, RFID system also suffers from security threats such as asynchronous attack, eavesdropping attack, forgery attack and so on. We compare the security performance of the new protocol with the scheme in the related literature mentioned above, as shown in Table 3 (Note: √ means resistible, × means irresistible, and O means unable to judge.).

## 6. Conclusions

In response to the existing problems in some current RFID authentication protocols and PUF application fields, we design a new type of PUF circuit structure based on the storage mechanism of RRAM. Compared with the known structure, this structure greatly expands the challenge response space and improves the computational efficiency. Based on the RRAM PUF, we propose an RFID authentication protocol, which uses multi-level PUF calculations and Hamming distance verification methods to achieve the encrypted transmission of data and reduce the amount of label calculations. Meanwhile, combined with RRAM PUF modeling and special error correction algorithms, the authentication reliability of the protocol is improved. The experimental results show that the protocol proposed in this paper can resist various known attacks and has good uniqueness and high reliability. At the same time, the reconfigurable feature of RRAM PUF will also greatly improve security.

## Figures and Tables

**Table 1:** Summary table of NIST test results.

Test	*p*-Value	Result
Frequency	0.834	Pass
Frequency within a Block	0.676	Pass
Runs Test	0.547	Pass
Longest Run of Ones in a Block	0.435	Pass
Binary Matrix Rank	0.541	Pass
Discrete Fourier Transform	0.427	Pass
Non-overlapping Template Matching	0.851	Pass
Overlapping Template Matching	0.744	Pass
Maurer’s Universal Statistical	0.472	Pass
Linear Complexity	0.624	Pass
Serial	0.784	Pass
Approximate Entropy	0.842	Pass
Cumulative Sums	0.745	Pass
Random Excursion	0.524	Pass
Random Excursion Variants	0.481	Pass

**Table 2:** Comparison of the RRAM PUF and the previous works.

	This Work	ISSCC’20 [28]	ISSCC’19 [29]	JSSC’18 [30]	TCAS-I’20 [31]
Type	RRAM	CMOS	CMOS	CMOS	CMOS
Bit Error Rate	$3.8\times 10^{-6}$	0.089%	0.30%	3.2%	0.04%
Inter HD	0.5001	0.4978	0.4998	0.4907	0.5032
Intra HD	0	-	-	0.0049	0.097

**Table 3:** Comparison of the RRAM PUF and the previous works.

Performance	Xie [24]	Zheng [25]	Sun [26]	Ye [27]	This Protocol
Physical attack	√	×	√	×	√
Eavesdropping attack	√	×	×	√	√
Forgery attack	√	√	√	√	√
Middleman attack	√	×	×	×	√
Replay attack	×	√	√	√	√
Nonsynchronous	√	×	√	√	√
Tracking attack	√	√	√	√	√
Machine learning	√	√	×	×	√
Reliability	O	×	O	O	√
